# An Engineered M13 Filamentous Nanoparticle as an Antigen Carrier for a Malignant Melanoma Immunotherapeutic Strategy

**DOI:** 10.3390/v16020232

**Published:** 2024-02-01

**Authors:** Nuša Brišar, Katja Šuster, Simona Kranjc Brezar, Robert Vidmar, Marko Fonović, Andrej Cör

**Affiliations:** 1 Faculty of Health Sciences, University of Primorska, 6310 Izola, Slovenia; nusa.brisar@fvz.upr.si; 2 Faculty of Medicine, University of Ljubljana, 1000 Ljubljana, Slovenia; 3 Valdoltra Orthopaedic Hospital, 6280 Ankaran, Slovenia; katja.suster@ob-valdoltra.si; 4 Department of Experimental Oncology, Institute of Oncology Ljubljana, 1000 Ljubljana, Slovenia; skranjc@onko-i.si; 5 Department of Biochemistry, Molecular and Structural Biology, Jozef Stefan Institute, 1000 Ljubljana, Slovenia; robert.vidmar@ijs.si (R.V.); marko.fonovic@ijs.si (M.F.); 6 Faculty of Education, University of Primorska, 6310 Izola, Slovenia

**Keywords:** nanoparticles, filamentous bacteriophages, phage display technology, bacteriophage-based vaccine, melanoma-associated antigen, malignant melanoma immunotherapy

## Abstract

Bacteriophages, prokaryotic viruses, hold great potential in genetic engineering to open up new avenues for vaccine development. Our study aimed to establish engineered M13 bacteriophages expressing MAGE-A1 tumor peptides as a vaccine for melanoma treatment. Through in vivo experiments, we sought to assess their ability to induce robust immune responses. Using phage display technology, we engineered two M13 bacteriophages expressing MAGE-A1 peptides as fusion proteins with either pVIII or pIIII coat proteins. Mice were intraperitoneally vaccinated three times, two weeks apart, using two different engineered bacteriophages; control groups received a wild-type bacteriophage. Serum samples taken seven days after each vaccination were analyzed by ELISA assay, while splenocytes harvested seven days following the second boost were evaluated by ex vivo cytotoxicity assay. Fusion proteins were confirmed by Western blot and nano-LC-MS/MS. The application of bacteriophages was safe, with no adverse effects on mice. Engineered bacteriophages effectively triggered immune responses, leading to increased levels of anti-MAGE-A1 antibodies in proportion to the administered bacteriophage dosage. Anti-MAGE-A1 antibodies also exhibited a binding capability to B16F10 tumor cells in vitro, as opposed to control samples. Splenocytes demonstrated enhanced CTL cytotoxicity against B16F10 cells. We have demonstrated the immunogenic capabilities of engineered M13 bacteriophages, emphasizing their potential for melanoma immunotherapy.

## 1. Introduction

Immunotherapy is a type of cancer treatment that uses the body’s immune system to fight against cancer. There are several types of cancer immunotherapy; among them, cancer vaccines have shown promising results, particularly in combination with other therapies. Therapeutic cancer vaccines are designed to prevent or treat cancer by targeting specific cancer cells or antigens, and provide long-term protection from recurrences and metastases [1]. Recent developments in cancer vaccines have led to a renewed interest in nanotechnology [2]. Bacteriophages, as nanoparticles, have been exploited in bacteriophage-display-based nanotechnology applications for new immunotherapeutic strategies [3].

Bacteriophages, or phages, are prokaryotic viruses that are the most abundant organisms on Earth and can be found in virtually every environment, including the human body. They infect bacteria by attaching to specific receptors on the surface of the bacterial cell, but they are generally considered to be harmless to human cells [4]. With many intriguing characteristics, bacteriophages have also become a promising tool in bioengineering [5]. Bacteriophages have been exploited for the development of powerful vaccine platforms in cancer immunotherapy, aiming to overcome the tolerogenic tumor microenvironment and, at the same time, to trigger specific immune responses [6]. Preclinical studies demonstrate the efficacy of bacteriophage-based vaccines against breast cancer [7], lung cancer [8], lymphoma [9], and melanoma [10]. They were also well tolerated and able to induce a clinical response in most patients with myeloma [11].

The path for bacteriophage engineering with phage display technology was paved by G. Smith. Phage display technology involves genetically engineering bacteriophages to display proteins or peptides in fusion with one of their capsid (i.e., coat) proteins [12]. A gene sequence encoding a peptide or protein of interest is cloned into a phage coat protein gene. Through the inherent machinery of the bacteriophage, one or more copies of the recombinant fusion proteins are synthesized and subsequently incorporated into the phage capsid and displayed on the surface of the bacteriophage [4]. The most widely and successfully exploited bacteriophages in phage display are filamentous bacteriophages [13]. M13 filamentous bacteriophages are non-lytic viruses with a unique rod-like protein cylinder composed of five coat proteins (pIII, pVI, pVII, pVIII, and pIX) [14]. Proteins pIII and pVIII are the most utilized for the display of peptides and differ in the number of peptides with which the capsid is decorated [13].

This study aimed to establish an engineered M13 filamentous bacteriophage displaying peptides of tumor-associated antigen MAGE-A1 on either pIII or pVIII as a potential vaccine treatment for malignant melanoma. Through in vivo experiments, we aimed to determine whether the bacteriophage vaccine can stimulate an immune response against the MAGE-A1 tumor antigen.

## 2. Materials and Methods

### 2.1. Construction of Recombinant MAGE Phagemids

Phagemids pComb8 (Plasmid#63889, Addgene, Watertown, MA, USA) and pComb3XSS (Plasmid#63890, Addgene, Watertown, MA, USA) were used for the construction of recombinant phagemids to display the MAGE-A1_161–169_ (EADPTGHSY) epitope on pVIII or pIII phage proteins, respectively. The pComb8 phagemid was first modified to remove the second cloning cassette by digestion with FastDigest Eco0109I and FastDigest EcoICRI (both Thermo Fisher Scientific, Waltham, MA, USA) at 37 °C for 10 min. Single-stranded extensions were removed using a Mung Bean Nuclease (New England Biolabs, Ipswich, MA, USA) treatment at 30 °C for 30 min, then self-circularization was performed using T4 DNA ligase (Thermo Fisher Scientific, Waltham, MA, USA). For cloning purposes, pComb8 was digested with FastDigest XhoI and FastDigest SpeI (both Thermo Fisher Scientific, Waltham, MA, USA) at 37 °C for 5 min, whereas pCom3XSS was digested with FastDigest SacI and FastDigest SpeI (both Thermo Fisher Scientific, Waltham, MA, USA) at 37 °C for 15 min, followed by a FastAP Thermosensitive Alkaline Phosphatase (Thermo Fisher Scientific, Waltham, MA, USA) treatment at 37 °C for an additional 10 min.

**Figure 1:**
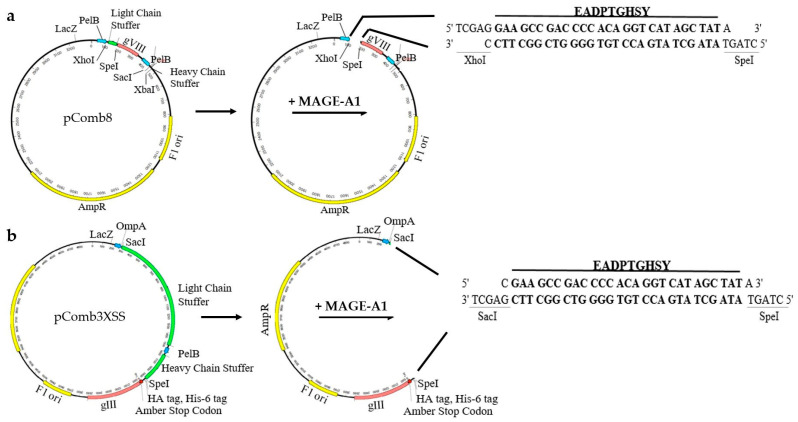
Schematic representation of the strategy used to construct recombinant phagemid (**a**) pComb8-MAGE- and (**b**) pComb3XSS-MAGE-expressing tumor peptide EADPTGHSY.

Recombinant MAGE phagemids were created via annealed oligonucleotide cloning. First, MAGE-A1_161–169_ fragments were created by annealing two overlapping 5′-phosphorylated oligonucleotides (Integrated DNA Technologies, IDT, Coralville, IA, USA): 5′-TCGAGGAAGCCGACCCCACAGGTCATAGCTATA-3′ and 5′-CTAGTATAGCTATGACCTGTGGGGTCGGCTTCC-3′ for cloning into the pComb8 vector, and 5′-CGAAGCCGACCCCACAGGTCATAGCTATA-3′ and 5′-CTAGTATAGCTATGACCTGTGGGGTCGGCTTCGAGCT-3′ for cloning into the pComb3XSS vector. Oligo annealing was performed with a QuantStudio 3 qPCR System (Applied Biosystems™, Waltham, MA, USA) by heating the suspension of oligonucleotides in equimolar concentrations (100 µM) to 95 °C over 4 min and gradually cooling down to 25 °C over 60 min. MAGE-A1 fragments were then ligated with backbone DNA fragments of digested pComb3XSS, 3342 bp, and pComb8, 3252 bp, using T4 DNA ligase at room temperature (RT) for one hour. The schematic representation of the construction of the recombinant phagemids is presented in Figure 1.

The ligated product was transformed into chemically competent *Escherichia coli* XL2—Blue cells (Agilent Technologies, Santa Clara, CA, USA) by exposing the cells to heat shock at 42 °C for 30 s. The suspension was plated on NZY agar plates (1% [*w*/*v*] NZ amine (Sigma Aldrich, St. Louis, MO, USA), 0.5% [*w*/*v*] yeast extract (Biolife, Milano, Italy), 0.5% [*w*/*v*] NaCl (Carlo Erba Reagents GmbH, Emmendingen, Germany), 1.25% [*v*/*v*] magnesium sulphate heptahydrate (Sigma Aldrich, St. Louis, MO, USA), 1.25% [*v*/*v*] magnesium chloride (Sigma Aldrich, St. Louis, MO, USA), 20% [*w*/*v*] glucose (Fisher Scientific, Waltham, MA USA)) supplemented with 50 µg/mL of carbenicillin (Fisher Bioreagents, Pittsburgh, PA, USA) and incubated at 37 °C overnight. The carbenicillin-resistant colonies were selected and cultured in LB broth (1% [*w*/*v*] tryptone (Biolife, Milano, Italy), 0.5% [*w*/*v*] yeast extract, and 1% [*w*/*v*] NaCl) containing 50 µg/mL carbenicillin overnight at 37 °C. Plasmids were isolated using the GenJET Plasmid Miniprep kit (Thermo Fisher Scientific, Waltham, MA, USA) according to the manufacturer’s instructions. The correct insertion of the MAGE-A1 peptide was determined via Sanger sequencing (Eurofins Genomics, Ebersberg, Germany) using the following primers (IDT, Coralville, IA, USA): pCom8-MAGE-Fw (5’-GCCGCAAATTCTATTTCAAGG-3’) and pCom8-MAGE-Rev (5’-GTAATACGACTCACTATAGG-3’), and pComb3XSS-MAGE-Fw (5’-GGAATTGTGAGCGGATAAC-3’) and pComb3XSS-MAGE-Rev (5’-CTCCTAAGAAGCGTAGTC-3’).

### 2.2. Genetically Engineered M13 Bacteriophage Production and Purification

A single transformant colony, harboring the recombinant MAGE phagemid, pComb8-MAGE or pCom3XSS-MAGE, was selected and inoculated in 3 mL 2 × YT broth (1.7% [*w*/*v*] tryptone, 1% [*w*/*v*] yeast extract, and 0.5% [*w*/*v*] NaCl) containing 1% (*w*/*v*) glucose, 50 μg/mL carbenicillin, and 40 μg/mL tetracycline (Sigma Aldrich, St. Louis, MO, USA) for overnight growth at 37 °C with shaking at 250 rpm on an MIR-S100 orbital shaker (SANYO Electric Co., Ltd., Osaka, Japan). The following day, 5 mL of 2 × YT broth containing 1% [*w*/*v*] glucose, 50 μg/mL carbenicillin, and 10 μg/mL tetracycline was inoculated with the overnight bacterial culture at a 1:200 ratio and incubated until the culture reached an OD600 of 0.5–0.6, at which point it was superinfected with the VCSM13 helper bacteriophage (Agilent Technologies, Santa Clara, CA, USA) at 10^12^ PFU/mL. The culture was incubated at 37 °C for 30 min without shaking and then for another 30 min with shaking. Bacteriophage-infected bacterial cells were pelleted via centrifugation in a Heraeus Multifuge 1 SR centrifuge (Heraeus, Hanau, Germany) for 10 min at 3300× *g* and 20 °C. The pellets were resuspended in 50 mL of 2 × YT broth containing 50 μg/mL carbenicillin and 50 μg/mL kanamycin, and shaken overnight at 37 °C. Supernatants containing genetically engineered bacteriophages were prepared via centrifugation for 15 min at 3000× *g* and 4 °C. Bacteriophages were precipitated on ice for 30 min with the addition of 4% [*w*/*v*] polyethylene glycol 6000 (PEG 6000; Merck KGaA, Darmstadt, Germany) and 3% [*w*/*v*] NaCl, followed by centrifugation at 15,000× *g* for 15 min at 4 °C. Bacteriophage pellets were resuspended in 0.5 mL 20 mM Tris-HCl, pH 8.0 (stock solution: 1 M Tris-HCl, pH 8.0; 12.11% [*w*/*v*] Tris Base (Fisher Scientific, Waltham, MA, USA); and 0.5% [*v*/*v*] HCl (Sigma Aldrich, St. Louis, MO, USA)). Residual bacterial debris was removed by a final centrifugation at 12,500× *g* for 10 min at 4 °C followed by filtration of the bacteriophage supernatant through a 0.2 µm syringe filter (Sartorius, Göttingen, Germany). The infective titers of genetically engineered bacteriophages were determined as described by Levisson et al. [15] and expressed as transforming units per mL (TFU/mL). Bacteriophages were stored at 4 °C until further use.

For animal experiments, additional purification processes for the bacteriophages displaying tumor peptides were optimized and carried out by JAFRAL d.o.o. At each purification step, the infective titer of phagemids in the bacteriophage suspensions and bacteriophage titer [15] was determined. The presence of endotoxins was determined by the chromogenic LAL test [16] and purified bacteriophages were diluted in PBS (137 mM NaCl, 8.1 mM Na_2_HPO_4_, 2.7 mM KCl, 1.5 mM KH_2_PO_4_, pH 7.4).

### 2.3. Detection of Recombinant Fusion Proteins by Western Immunoblotting and Nano LC-MS/MS Analysis

Bacteriophage samples (10^12^ PFU) were diluted in 6× SDS sample buffer (Thermo Fisher Scientific, Waltham, MA, USA) and heated at 97 °C for 3 min. The obtained protein supernatants were separated on a 12.5% SDS-PAGE gel (Lonza, Basel, Switzerland). To confirm the expression of recombinant copies of pIII with a fused MAGE-A1 epitope, Western blot analysis was performed, whereas for the confirmation of pVIII with fused MAGE-A1 epitope, nano LC-MS/MS analysis had to be performed due to the fused peptide’s small size.

For Western immunoblotting, separated proteins were electroblotted onto a nitrocel-lulose membrane (Serva Electrophoresis GmbH, Heidelberg, Germany). The blotted membrane was blocked in 5% skim milk in TBST (TBS: 20 mM Tris, pH 7.6, 150 mM NaCl with 0.1% Tween-20 (Sigma Aldrich, St. Louis, MO, USA)) under agitation for one hour at RT. For the detection of fusion proteins, 6× His Tag Monoclonal Antibody (dilution 1:800, 4E3D10H2/E3, Invitrogen, Waltham, MA, USA) was incubated with the membrane overnight at 4 °C, under agitation. After four washes in TBST (15 min each), secondary Peroxi-dase AffiniPure Goat Anti-Mouse IgG + IgM (H + L) antibodies (dilution 1:5000, 115-035-068, Jackson Immunoresearch, Cambridgeshire, UK) were added to the membrane and incubated under agitation for one hour at RT. After four washes (15 min each) in TBST, an ECL kit (GE Healthcare, Chicago, IL, USA) was used for detection. The immunoreactive bands were then visualized using a chemiluminescent detection system, the UVP ChemStudio PLUS imager gel documentation system (Analytik Jena, Jena, Germany).

For mass spectrometry analysis, proteins separated on an SDS-PAGE gel were stained with Coomassie Brilliant Blue (Serva Electrophoresis GmbH, Heidelberg, Germa-ny). A protein lane 10 kDa in size was cut and further prepared for mass spectrometry as previously described [17]. LC-MS/MS analyses were performed with an EASY-nano LC II HPLC unit (Thermo Scientific, San Jose, CA, USA) coupled to an Orbitrap LTQ Velos mass spectrometer (Thermo Scientific, San Jose, CA, USA). MS/MS spectra were obtained by the higher energy collision dissociation fragmentation (normalized collision energy at 35) of the nine most intense precursor ions from the full MS scan. Data analysis was performed as described by Sobotič et al. [17]. Briefly, analysis of the database search and quantification via spectral counting were performed using the MaxQuant proteomics software (version 2.0.30), with an embedded Andromeda search engine. The search was performed against the *E. coli* and bacteriophage M13 proteome database in UniProt, using the trypsin cleavage specificity with a maximum of two missed cleavages. The carbamidomethylation of cysteine was static, whereas methionine oxidation and N-terminal acetylation were dynamic modifications.

### 2.4. Vaccination of Mice for Sera Analyses and Cytotoxicity Assay

**Figure 2:**
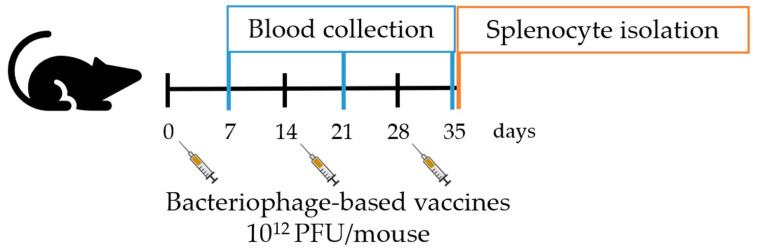
Schematic representation of in vivo experiments.

The experiments performed in this study complied with the guidelines for animal experiments from the EU directive (2010/63/EU) and with the permission of the Administration of the Republic of Slovenia for Food Safety, Veterinary and Plant Protection (Republic of Slovenia, Ministry of Agriculture, Forestry and Food, permission no. U34401-3/2022/11) and were in accordance to the 3R principle. The animals used were 6–8-week-old female C57BL/6NCrl mice (Charles River, Lecco, Italy). Mice were maintained in specifically pathogen-free conditions in a carousel mouse IVC rack system (Animal Care Systems Inc., Centennial, CO, USA) at 20–24 °C, 55 ± 10% humidity, and with a 12 h light/dark cycle. Food and water were provided ad libitum. Mice (5 mice per group/cage) were injected intraperitoneally three times at consecutive two-week intervals (as shown in Figure 2) with 300 µL of: (1) genetically engineered pVIII::MAGE-A1_161–169_ bacteriophages (10^12^ PFU), (2) genetically engineered pIII::MAGE-A1_161–169_ bacteriophages (10^12^ PFU), (3) wild-type M13 bacteriophages (10^12^ PFU), or (4) PBS. Sera (50 µL) for ELISA assays were obtained from blood samples collected with a sterile capillary from a retro-orbital sinus one week after each boost and stored at −80 °C until use. For the ex vivo spleen cytotoxicity assay, splenocytes were collected from aseptically removed spleens one week after the last bacteriophage application and prepared as described by Komel et al. [18].

For the ex vivo cytotoxicity assay, B16F10 murine melanoma target cells (American Type Culture Collection, Manassas, VA, USA) (2.5 × 10^7^ cells/mL) were incubated with 5 μM of the fluorescent cell dye Carboxyfluorescein succinimidyl ester (CFSE; Biolegend, San Diego, CA, USA) following the manufacturer’s instructions. Then, cells were centrifuged at 475× *g* for 5 min and pelleted cells were resuspended in advanced Dulbecco’s Modified Eagle Medium (DMEM) (Gibco, Waltham, MA, USA), supplemented with 5% [*v*/*v*] fetal bovine serum (FBS; Gibco, Waltham, MA, USA), 10 mL/L L-glutamine (Gluta-MAX; Gibco, Waltham, MA, USA), and 10 mL/L Penicillin–Streptomycin (Gibco, Waltham, MA, USA). Resuspended cells were seeded in a black-tissue-culture-treated 96-well microplate (Greiner Bio-One, Kremsmünster, Austria) at a density of 354 cells per well and incubated overnight. The next day, target cells were incubated with thawed viable splenocytes at an effector: target ratio of 50:1 in Roswell Park Memorial Institute (RPMI) 1640 medium (Gibco, Waltham, MA, USA) supplemented with 5% [*v*/*v*] FBS, 10 mL/L L-glutamine, and 10 mL/L Penicillin–Streptomycin, in the dark for 48 h. Splenocytes were restimulated with IL-2 (0.02 ng/µL per well) (Miltenyi Biotec, Bergisch Gladbach, Germany). Fluorescent cells were counted at λex = 492 nm and λem = 517 nm using the BioTek Cytation 1 Cell Imaging Multimode Reader (BioTek, Winooski, VT, USA). Images of labelled tumor cells were captured using a 4× objective and GFP (469/525) imaging filter cube; the quantification of labelled cells and calculation of specific survival in co-culture and control wells were performed as described in [18].

The amount of antibodies in mice sera against wild-type M13 bacteriophages and MAGE-A1_161–169_ peptides was determined by the ELISA assay. Nunc MaxiSorp™ 96-well plates (Thermo Fisher Scientific, Waltham, MA, USA) were coated overnight at 4 °C with (1.) 100 μL VCSM13 bacteriophages (10^7^ PFU) in carbonate coating buffer (Thermo Fisher Scientific, Waltham, MA, USA), (2.) 100 µL MAGE-A1 peptides (ref. number A7187-1, Thermo Fisher Scientific, Waltham, MA, USA) (10 µg/mL) in coating buffer, or (3.) 100 µL PBS (for negative control). Plates were washed three times with PBS containing 0.1% Tween-20 (PBST) and blocked with eBioscience™ ELISA/ELISPOT diluent (1×) (Invitro-gen, Waltham, MA, USA) for 1 h at RT. Before the addition of serum samples, wells were washed two times with PBST. The dilution rate of serum samples in 1× PBS was 1:20,000 for the detection of anti-M13 antibodies, and 1:6000 for the detection of antibodies against MAGE-A1_161–169_ peptides. After the addition of diluted serum to the wells, the plate was incubated for 2 h at RT. Plates were then washed three times with PBST, and, for the detection of bound antibodies, HRP labelled goat anti-mouse IgG, IgM (H + L), and highly cross-adsorbed secondary antibody (dilution 1:1000, SAB3700986, Sigma-Aldrich, St. Louis, MO, USA) were added and incubated for 1 h at RT. Finally, the plates were washed four times with PBST and, for detection, eBioscience™ TMB Solution (1×) (Thermo Fisher Scientific, Waltham, MA, USA) was added for 15 min at RT. The reaction was stopped by the addition of the Stop solution (Thermo Fisher Scientific, Waltham, MA, USA) and the OD450 was measured using the BioTek Cytation 1 Cell Imaging Multimode Reader (BioTek, Winooski, VT, USA).

For the immunohistochemical analyses, B16F10 cells were fixed with 4% paraform-aldehyde and incubated with 100 μL of mouse serum (dilution 1:50) overnight. HRP la-belled goat anti-mouse IgG, IgM (H + L), highly cross-adsorbed secondary antibody (dilu-tion 1:1000, SAB3700986, Sigma-Aldrich, St. Louis, MO, USA), and AEC Substrate (Abcam, Cambridge, UK), as the colorogenic reagent, were used. Imaging was performed using a BX-51 microscope (Olympus, Hamburg, Germany) equipped with a digital camera (DP72; Olympus, Hamburg, Germany).

### 2.5. Statistical Analysis

Statistical analyses and graphical presentations were performed using GraphPad Prism software 10.1.2. (GraphPad Software, Boston, MA, USA). Data were tested for nor-mal distribution using the Shapiro–Wilk test. Group differences in the ELISA assay were analyzed using a two-way analysis of variance (ANOVA), followed by Uncorrected Fisher’s LSD and Tukey’s multiple comparisons test for group comparisons. Group differences in the ex vivo spleen cytotoxicity assay were analyzed using a one-way ANOVA, followed by Uncorrected Fisher’s LSD test for group comparisons. Statistical significance was defined as *p* < 0.05. Data are presented as the arithmetic mean (AM) and standard error of the mean (SEM).

## 3. Results

### 3.1. The Construction of Phagemids and Identification of Bacteriophage-Displayed Tumor Peptides

The Sanger sequencing of the constructed phagemids confirmed the successful construction of pComb3XSS-MAGE and pComb8-MAGE. The engineered pComb3XSS-MAGE’s sequence revealed truncated pIII fused to MAGE epitope’s sequence and a His tag, whereas pComb8-MAGE’s sequence showed the MAGE epitope’s sequence fused to a truncated pVIII sequence.

After the transformation of *E. coli* bacterial cells with the recombinant phagemids pComb8-MAGE or pComb3XSS-MAGE followed by super-infection with VCSM13 helper bacteriophages, genetically engineered M13 bacteriophages were released.

To confirm the expression of recombinant copies of pIII with a fused MAGE-A1 epitope, Western blot analysis was performed. An immunoreactive protein band with a molecular weight of approximately 25 kDa was obtained. This band was regarded as the fusion protein of truncated pIII (18.23 kDa), HA tag (1.1 kDa), His tag (0.8 kDa), and the MAGE-A1_161–169_ tumor peptide (975.9 Da), encoded by phagemid pCom3XSS-MAGE’s genome. No reactive band was detected in the control lane to which a wild-type pIII of the VCSM13 helper bacteriophage had been blotted (Figure S1).

Nano LC-MS/MS analysis was performed to confirm the expression of recombinant copies of pVIII with a fused MAGE-A1 epitope from the phagemid pComb8-MAGE’s genome. A protein lane corresponding to approximately 10 kDa was cut from the SDS-PAGE gel for verification via N-terminal amino acid sequencing. The fusion protein pVIII-MAGE-A1 was expected to correspond to a molecular weight of 6.23 kDa, truncated pVIII to 5.24 kDa, and the MAGE-A1_161–169_ tumor peptide to 975.9 Da. To find the closest match, experimentally obtained spectra were compared with the theoretical spectra generated in silico from the proteome database UniProt data of the M13 bacteriophage with fusion protein pVIII-MAGE-A1. Based on the obtained peptide sequences AEGDDPAK and SYTSAEGDDPAK, the fusion protein pVIII-MAGE-A1 was identified by four of its amino acids: SYTS. According to the coverage of b and y ions observed in the MS/MS spectrum of peptide SYTSAEGDDPAK (Figure S2), the successful fusion of the tumor peptide with pVIII was predicted. Sequence coverage was also analyzed against the proteome database UniProt data of the wild-type M13 bacteriophage and the following five peptides were identified: AEGDDPAK, FAAEGDDPAK, LSFAAEGDDPAK, MLSFAAEGDDPAK, and SFAAEGDDPAK. According to their score value, pVIII was predominantly expressed in the wild-type form encoded by the VCSM13 genome, interspersed with recombinant fusion proteins (Table 1).

### 3.2. Ex Vivo Spleen Cytotoxicity Assay

**Figure 3:**
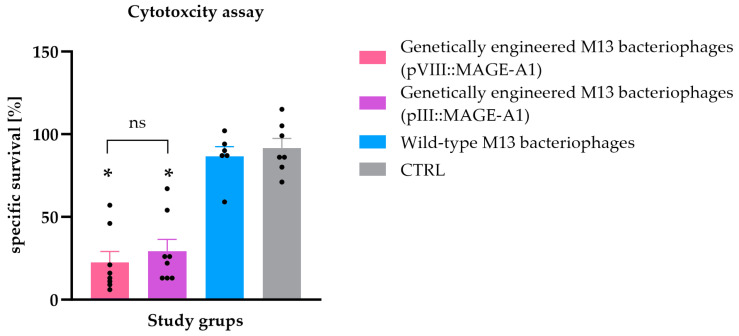
Ex vivo spleen cytotoxicity assay. The specific survival rate (%) of B16F10 malignant melanoma cells after incubation with splenocytes isolated from C57BL/6NCrl mice immunized with genetically engineered M13 bacteriophages (pVIII::MAGE-A1), genetically engineered M13 bacteriophages (pIII::MAGE-A1), wild-type M13 bacteriophages, and from the CTRL group. Legend: *, *p* < 0.05; ns, not statistically significant. The values are presented as the AM ± SEM. Repeated measurements are represented by black dots.

To assess the cytotoxic activity of the immune cells of vaccinated animals, an ex vivo spleen cytotoxicity assay was performed. B16F10 tumor cells incubated with splenocytes recovered from mice vaccinated with the genetically engineered M13 bacteriophages had a statistically significant lower survival rate compared to B16F10 tumor cells incubated with splenocytes from mice immunized with wild-type M13 bacteriophages and the CTRL group (*p* ≤ 0.05). The difference in the cytotoxic activity of splenocytes from mouse groups vaccinated with genetically engineered M13 bacteriophages displaying either the pVIII::MAGE-A1 or pIII::MAGE-A1 fusion proteins was not statistically significant (Figure 3).

### 3.3. Evaluation of the Antibody Response

To assess the potential activation of humoral immunity by wild-type bacteriophage nanoparticles and/or genetically engineered bacteriophage nanoparticles, sera from vaccinated mice were screened for the presence of anti-M13 bacteriophage and anti-MAGE antibodies using an ELISA assay.

**Figure 4:**
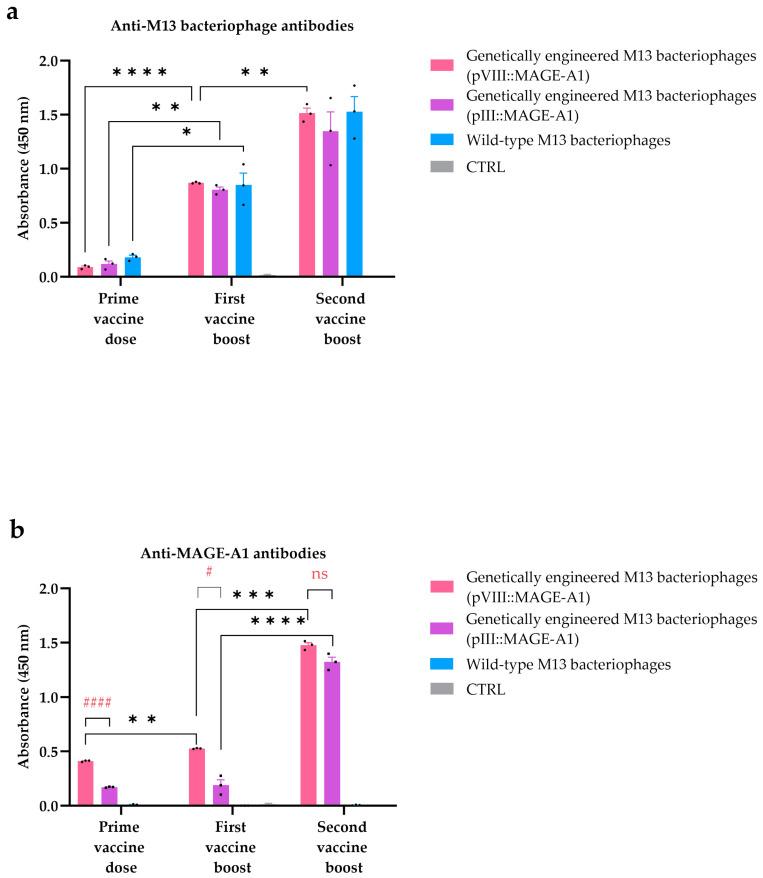
ELISA assay for the detection of (**a**) anti-M13 bacteriophage and (**b**) anti-MAGE antibodies in mouse sera. The antibody response was assessed in C57BL/6NCrl mice immunized with genetically engineered M13 bacteriophages (pVIII::MAGE-A1), genetically engineered M13 bacteriophages (pIII::MAGE-A1), wild-type M13 bacteriophages, and in the CTRL group. Legend: *: *p* ≤ 0.05, **: *p* ≤ 0.01, ***: *p* ≤ 0.001, ****: *p* ≤ 0.0001, values were considered statistically significant for the comparison of immune responses across different vaccine doses; #: *p* ≤ 0.05 and ####: *p* ≤ 0.0001 were considered statistically significant for the comparison of immune responses between the genetically engineered M13 bacteriophages (pVIII::MAGE-A1) and genetically engineered M13 bacteriophages (pIII::MAGE-A1) groups within one vaccine dose; ns: not statistically significant. The values are presented as the AM ± SEM. Repeated measurements are represented by black dots.

Anti-M13 bacteriophage antibodies were found in all the tested sera from mice vaccinated with any type of bacteriophages, with their levels increasing after each administered dose. Anti-M13 bacteriophage antibody levels in the group vaccinated with genetically engineered M13 bacteriophages displaying pVIII::MAGE-A1 were statistically significantly higher in the first vaccine boost compared to the prime vaccine dose (first vaccine boost: average measurement 0.870, prime vaccine dose: average measurement 0.090, *p* ≤ 0.0001) and in the second vaccine boost compared to the first vaccine boost (second vaccine boost: average measurement 1.514, first vaccine boost: average measurement 0.870, *p* ≤ 0.01). Similarly, anti-M13 bacteriophage levels were higher when comparing the first vaccine boost to the prime vaccine dose in the group vaccinated with genetically engineered M13 bacteriophages displaying pIII::MAGE-A1 (*p* ≤ 0.01) and in the group vaccinated with wild-type bacteriophages (*p* ≤ 0.05). However, in these two groups, a statistically significant difference was not observed between the second and first vaccine boost. Anti-M13 bacteriophage antibodies were not found in the sera of control mice group immunized with PBS (CRTL group) (Figure 4a).

Sera from mice vaccinated with genetically engineered M13 bacteriophages display-ing the pVIII::MAGE-A1 or pIII::MAGE-A1 fusion proteins demonstrated the presence of anti-MAGE antibodies, confirmed by the binding of these antibodies to synthetic MAGE-A1_161–169_ peptides. The anti-MAGE levels in the group vaccinated with genetically engineered M13 bacteriophages displaying pVIII::MAGE-A1 were statistically significantly higher in the first vaccine boost compared to the prime vaccine dose (first vaccine boost: average measurement 0.527, prime vaccine dose: average measurement 0.411, *p* ≤ 0.01) and in the second vaccine boost compared to the first vaccine boost (second vaccine boost: average measurement 1.476, first vaccine boost: average measurement 0.527, *p* ≤ 0.001). A statistical significance in anti-MAGE levels was also observed between doses of engineered M13 bacteriophages displaying pIII::MAGE-A1, but only when comparing the second vaccine boost to the first vaccine boost (*p* ≤ 0.0001). When comparing the anti-MAGE levels between the genetically engineered M13 bacteriophages (pVIII::MAGE-A1) and genetically engineered M13 bacteriophages (pIII::MAGE-A1) groups, a significant difference was evident only in the prime vaccine dose (*p* ≤ 0.0001) and the first vaccine boost (*p* ≤ 0.05). However, no significant difference in anti-MAGE levels between these two treatment groups was observed after the second vaccine boost (*p* > 0.05). No anti-MAGE antibodies were found in the sera of the wild-type bacteriophage vaccinated group or the CRTL group (Figure 4b).

**Figure 5:**
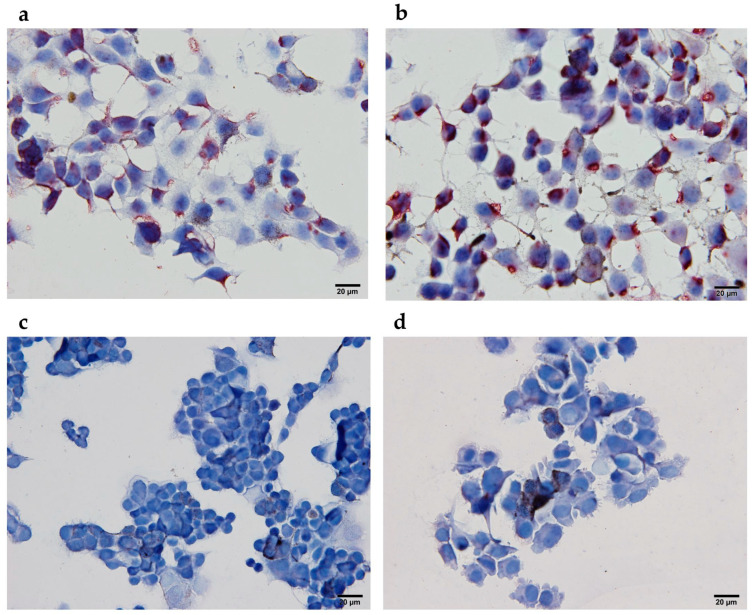
Immunocytochemical analyses of B16F10 melanoma cells expressing MAGE-1_161–169_ tumor epitopes after incubation with sera from (**a**) genetically engineered M13 bacteriophages (pIII::MAGE-A1), (**b**) genetically engineered M13 bacteriophages (pVIII::MAGE-A1), (**c**) wild-type M13 bacteriophages, and (**d**) in the CTRL group. Red staining represents the expression of MAGE-1_161–169_ tumor epitopes.

To further exploit the binding ability of specific anti-MAGE antibodies to naturally expressed tumor epitopes, immunocytochemical analyses of B16F10 tumor cells were performed after their incubation with pooled mice sera. As expected, only the blue staining of nuclei and no red staining of tumor cells was observed after incubation with sera from mice vaccinated with wild-type M13 bacteriophages or those in the CTRL group. On the other hand, anti-MAGE antibodies, present in the sera from mice vaccinated with genetically engineered M13 bacteriophages displaying pVIII::MAGE-A1 or pIII::MAGE-A1 fusion proteins, were able to bind to the MAGE-A1_161–169_ tumor epitopes present on tumor cells, which resulted in their staining (Figure 5).

## 4. Discussion

In the present study, we describe the preparation of two types of genetically engineered M13 filamentous bacteriophage virion surfaces decorated with either an pIII or pVIII coat protein fused to peptides derived from melanoma-associated antigen MAGE-A1_161–169_. Through an in vivo study, we have shown that administering an M13 filamentous bacteriophage-based vaccine in mice induces an anti-MAGE-A1 antibody response and generates antigen-specific cytotoxic T lymphocytes (CTLs) capable of targeting and eliminating tumor cells.

The MAGE antigen was chosen because it is expressed in several malignancies, including 48% of metastatic melanomas, but not in normal tissues, except in male germinal cells and the placenta [19]. Studies support the involvement of MAGE-As in the process of oncogenesis, metastasis, and the survival of cancer cells. This makes them a compelling candidate for the development of targeted and efficient immunotherapeutic vaccination approaches [20,21]. Several MAGE peptide-based vaccine therapies have been developed for clinical applications which have resulted in a delayed immune response and tumor growth inhibition; however, they do not demonstrate substantial tumor shrinkage [22]. Peptides on their own have a low immunogenicity profile and a short lifetime [23]. Soluble antigens fail to enter the appropriate intracellular compartment for MHC I presentation [24]. To enhance the efficacy of peptide-based vaccines pharmaceutical formulations incorporating immune-stimulating adjuvants have been employed [25]. Jiang et al. [26] discovered that mice immunized with the MAGE-1Hsp70 fusion protein exhibited markedly elevated titers of MAGE-1-specific antibodies, more robust CTLs, and the increased secretion of IFN-γ compared to mice immunized with MAGE-A1 alone. To deliver MAGE-A1 in a highly immunogenic form, aiming to induce a specific immune response while prolonging the epitope’s persistence and half-life, a bacteriophage carrier was selected [27].

Different lytic bacteriophages, such as lambda [28,29], T7 [30,31], and T4 [32,33,34] have been proposed for use as delivery systems for proteins and peptides in cancer diagnosis and treatment. In this study, M13 filamentous bacteriophages were chosen over their lytic counterparts for their non-lytic nature, facilitating their easier production and purification [35].

Recognized as foreign antigens, filamentous bacteriophages per se mediate an immunogenic response by stimulating innate and adaptive immunity towards bacteriophage-displayed peptides. Due to their single-stranded DNA rich in CpG motifs, they can directly activate the Toll-like receptors’ (TLRs) innate immune pathway to further induce adaptive immune responses. M13 bacteriophages are internalized by antigen-presenting cells (APCs) as inert antigen particles, facilitating antigen release and cross-presentation for the subsequent activation of CD4+ (MHC II) and CD8+ (MHC I) T-cell responses [36].

We have shown that M13 filamentous bacteriophages with MAGE-A1 fused with pIII or pVIII proteins generate a CTLs response capable of killing tumor cells. Initial investigations on the T-cell responses to the peptides MAGE-A3_271–279_ and MAGE-A10_254–262_, when presented by dendritic cells, have demonstrated that eliciting specific CTL responses necessitated recurrent stimulations in vitro and that repeated immunizations seldom elicited CTL responses in vivo [37,38,39]. The efficacy of bacteriophage-mediated APC cross-presentation is superior to that of free antigens. This enhanced efficacy includes increased cellular uptake and higher levels of immunogenicity [40]. In the present study, we evaluated the induction of cell-mediated immunity via an ex vivo cytotoxicity assay. Based on the results, we infer that vaccinating mice with genetically engineered bacteriophages induced potent CTLs, which, upon in vitro restimulation, identified the low antigen levels on B16F10 tumor cells and initiated their lysis (Figure 3). It is important to note that this experimental approach indirectly demonstrated the presence of specific CTLs within the splenocytes. However, the use of H-2Kb/MAGE-A1 multimers may provide direct evidence of tumor-specific cytotoxic T cells and their presence in the splenocytes isolated from immunized mice [41]. A study by Wang et al. [42] also used M13 bacteriophages, but in a breast cancer model. Their research also showed that M13 bacteriophages displaying HER2-induced T-cell cytotoxicity. Similar to our study, this was confirmed in vitro by the incubation of splenocytes from immunized mice with a Δ16HER2-positive breast cancer cell line. In two to other studies [43,44], similar results were obtained using another type of filamentous bacteriophage—the *fd* bacteriophage. In a study by Sartorius et al. [43], the authors also used a MAGE antigen, more precisely MAGE-A3_271–279_ or MAGE-A10_254–262_, fused to the pVIII protein. A strong anti-tumor CTL was observed in splenocytes from mice vaccinated with the *fd* phage displaying p23 and one of the selected tumor peptides fused to the pVIII. Similarly, splenocytes from mice vaccinated with *fd* phages displaying P1A_35–43_, a murine protein that shares several characteristics with human MAGEs, fused to the pVIII, showed specific anti-P1A cytotoxic activity against murine mastocytoma P815 [44]. In a study conducted by Fang et al. [10], splenocytes from mice immunized with the engineered bacteriophage displaying a MAGE peptide fused to pVIII exhibited specificity in recognizing the MAGE-A1_161–169_ peptides and displayed varying lytic activity against B16F10 cells at different effector-to-target ratios. In contrast, mice immunized with wild-type M13 bacteriophages or PBS did not exhibit MAGE-specific cytotoxicity, which is consistent with the outcomes observed in our investigation. Fang’s study assessed cytotoxicity using a standard Chromium release assay. In our study, we followed the methodology outlined by Komel et al. [3]. Tumor cells were fluorescently labeled, and their lysis was measured 48 h after the addition of bacteriophages. Upon comparing our results with their study, we observed that engineered bacteriophages exhibited cytotoxicity as effectively as the gene electrotransfer (GET) of plasmids encoding IL-2 and IL-12, albeit in a different tumor model. These data suggest that using filamentous bacteriophages as vaccine carriers has the advantage of inducing a cellular response, a key feature of anticancer vaccines.

Through an ELISA assay, we have further validated the activation of humoral immunity, underscoring the crucial role of the repeated display of MAGE peptides on the surface of filamentous nanoparticles for improved B cell responses (Figure 4b). Additionally, with immunocytochemistry, we showed that bacteriophage-induced anti-MAGE antibodies can also bind to naturally expressed MAGE-A1 epitopes on the surface of B16F10 cancer cells in vitro (Figure 5). In a study by Bartolacci et al. [45], the author constructed ∆16HER2, a splicing variant of Her2 in breast cancer fused to a minor coat protein. Similarly, using an ELISA assay, they showed that the engineered M13 bacteriophage effectively induced anti-∆16HER2 antibodies. Notably, in an in vivo experiment, these antibodies even broke immune tolerance to the HER2 self-antigen and induced protective immunity in a mouse model of breast cancer.

Wild-type bacteriophages showed a lack of affinity for binding to the MAGE antigen (Figure 4b). In a similar manner, Murgas et al. [46] demonstrated the display of a single-chain variable fragment (ScFv), specific to CEA (carcinoembryonic antigen, highly expressed in colorectal cancer), on pIII. Using an ELISA assay, they showed that the wild-type phage showed no binding, whereas the engineered bacteriophages effectively bound to the CEA protein.

While the emergence of anti-bacteriophage antibodies (Figure 4a) could be a concern in human therapy, potentially diverting the antibody response away from the intended target, the peculiar structure of filamentous bacteriophages contains few endogenous B cell epitopes; therefore, we concluded that this would not be a significant issue. Also, Van Houten et al. [47] showed that modifications to bacteriophages reduce the intrinsic immunogenicity of the bacteriophage coat protein, thereby directing the antibody response mainly towards the exogenous (poly)peptides displayed on engineered virions. The limited presence of endogenous B cell epitopes may be also attributed to the co-evolution between the filamentous bacteriophage and the human body. The bacteriophage only infects enterobacterium *E. coli* carrying an F’ episome. To survive in the human gut, they have evolved proteins that trigger a mild B cell response, helping them partly avoid the human body’s mucosal antibody defense [35].

Previous research indicated that the dosing protocol influenced the titer and affinity of the induced antibodies [48]. In our study, we explored engineered M13 bacteriophages with a pIII display (pIII::MAGE-A1) and engineered M13 bacteriophages with a pVIII display (pVIII::MAGE-A1) to investigate whether the display method significantly affects adaptive immunity. The vaccines employing phage display were previously designed on either the pVII or pIII protein [10,42,44,45,46,49]. The existing literature lacks a comparative assessment of these two approaches. Displaying an epitope in fusion with the major coat protein (pVIII) allows for high display densities with up to a thousand copies of the immunogenic tumor peptides out of 2700 copies of the pVIII protein, and is more effective at generating high antibody titers compared to the same epitope displayed in a low copy number on minor coat protein (pIII) [50]. According to the literature, when using a pIII display, approximately 10% of phages will exhibit one fifth of the fusion proteins out of a total of five copies of the pIII coat protein. A smaller proportion of phages will display two or more fusion proteins, while the majority of phages will not display any [51]. We observed that valency (tumor peptide copies per phage) is related to the generation of anti-tumor peptide antibodies after the initial prime dose and the first booster dose. However, the difference between the displays diminished after the second booster dose. Based on our results, we can infer that after multiple vaccinations with bacteriophages, a plateau in the humoral immune response is reached, along with the quantity of obtained antibodies. Similarly, no statistically significant difference in cytotoxicity was observed in CTLs harvested after the third vaccine dose that specifically targeted B16F10 tumor cells. Hence, it can be inferred that the selection of the display method is primarily contingent on the type of (poly)peptide intended for display. As evident from the literature, high display valences of a pVIII display carry the risk of steric effects that may impair or slow phage assembly [50]. When wanting to produce a phage construct displaying the whole tumor antigen or its domains, a viable solution is to fuse it to five copies of the minor coat protein pIII and increase the number of vaccinations [42]. However, for a single or double vaccination, the presentation of pVIII offers an advantage in reducing the stress on experimental animals, although this is limited to the display of peptides [52].

We acknowledge that the limitation of our study is that we did not thoroughly investigate the MAGE-derived H-2Kb-restricted CTL epitopes in existing databases. Further investigation of this area, possibly using H-2Kb/Mage-A1 multimers, could provide direct evidence of the presence of tumor-specific CTLs in splenocytes isolated at a specific time point after therapy.

Combining a relatively simple structure with endogenous adjuvant activity and the powerful ability to insert foreign genes and display foreign proteins/peptides on its surface, a M13 bacteriophage-based bionanomaterial is a compelling delivery platform that can be used to develop effective and safe anticancer therapies. Further studies, to the determine preventive and curative effects against melanoma in vivo and to assess the tumor microenvironment conditions and how they interact with engineered nanoparticles, would be interesting to conduct in the future. Large clinical trials are also required to fully establish bacteriophages’ safety in humans, as well as to optimize their vaccine design.

## 5. Conclusions

We demonstrated the possibility of inducing specific anti-MAGE antibodies and CTL cytotoxicity through the intraperitoneal administration of MAGE-A1 peptides displayed on engineered non-lytic M13 bacteriophages. This underscores the potential of filamentous bacteriophages in advancing cancer immunotherapy. The M13 bacteriophage, acting as a versatile carrier, facilitates the swift adaptation of new epitopes to diverse cancer-type antigens. The observed immunogenicity of the prepared M13 bacteriophage nanoparticles warrants further exploration, whether as an independent therapeutic modality or in synergistic conjunction with existing conventional cancer treatments.

## Figures and Tables

**Table 1:** Amino acid sequences of the wild-type pVIII and the fusion protein pVIII-MAGE-A1.

Bacteriophage Protein	Amino Acid Sequence
Wild-type pVIII	MKKSLVLKASVAVATLVP**MLSFA****AEGDDPAK**AAFNSLQASATEYIGYAWAMVVVIVGATIGIKLFKKFTSKAS
pVIII-MAGE-A1	*LEEADPTGH**SYTS*****AEGDDPAK**AAFNSLQASATEYIGYAWAMVVVIVGATIGIKLFKKFTSKAS

Bold, identified peptides; underlined, difference between modified pVIII compared to the wild-type pVIII; italic, tumor peptide sequence with enzyme restriction sites.

## Data Availability

The data presented in this study are available on request from the corresponding author.

## Supplementary Materials

The following supporting information can be downloaded at https://www.mdpi.com/article/10.3390/v16020232/s1, Figure S1: Western immunoblotting of the fusion protein pIII::MAGE-A1 separated by reducing its SDS-PAGE; Figure S2: MS/MS spectra of the identified peptide SYTSAEGDDPAK via nano LC-MS/MS.
