# Supplementary material for: An Engineered M13 Filamentous Nanoparticle as an Antigen Carrier for a Malignant Melanoma Immunotherapeutic Strategy
Source: Viruses. 2024 Feb 1;16(2):232. doi: 10.3390/v16020232 (PMC10893169; doi:10.3390/v16020232)
Supplement: Supplementary file 1 [file viruses-16-00232-s001.zip › viruses-2846109-supplementary.pdf]

Supplementary Materials

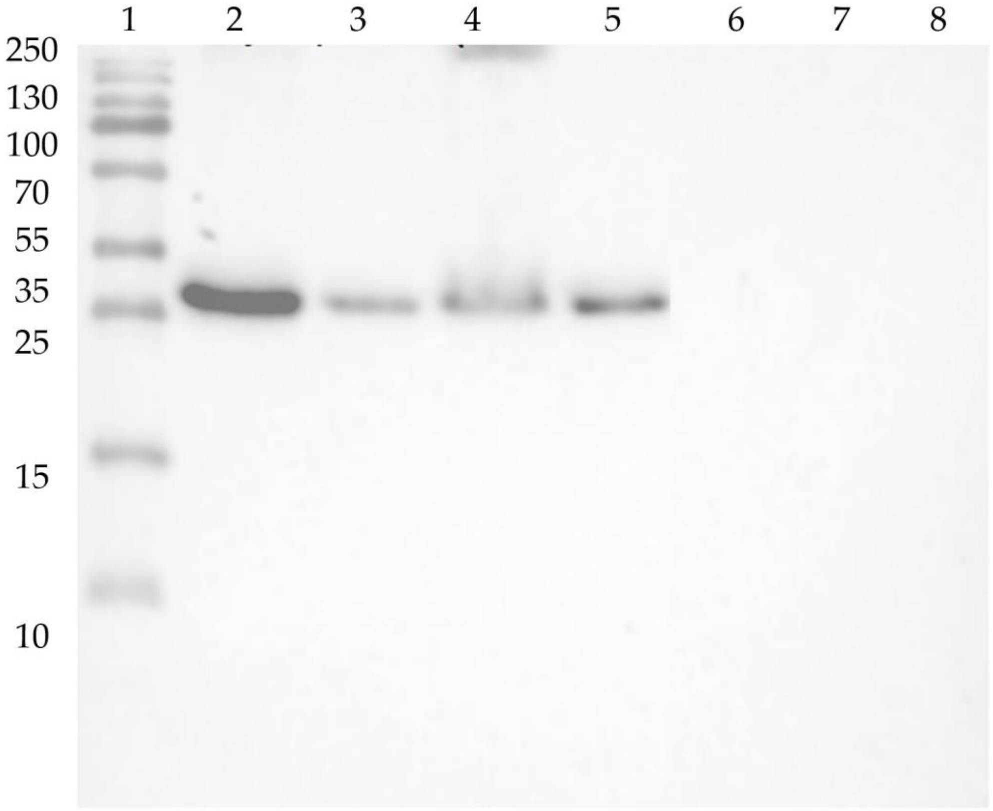

Figure S1. Western immunoblotting of fusion protein pIII::MAGE-A1 separated by reducing SDS-PAGE. Lane 1: molecular weight markers in kDa, lines 2-5: fusion protein pIII::MAGE-A1 of genetically engineered bacteriophages, lines 6-8: wild-type pIII of VCSM13 helper bacteriophage.

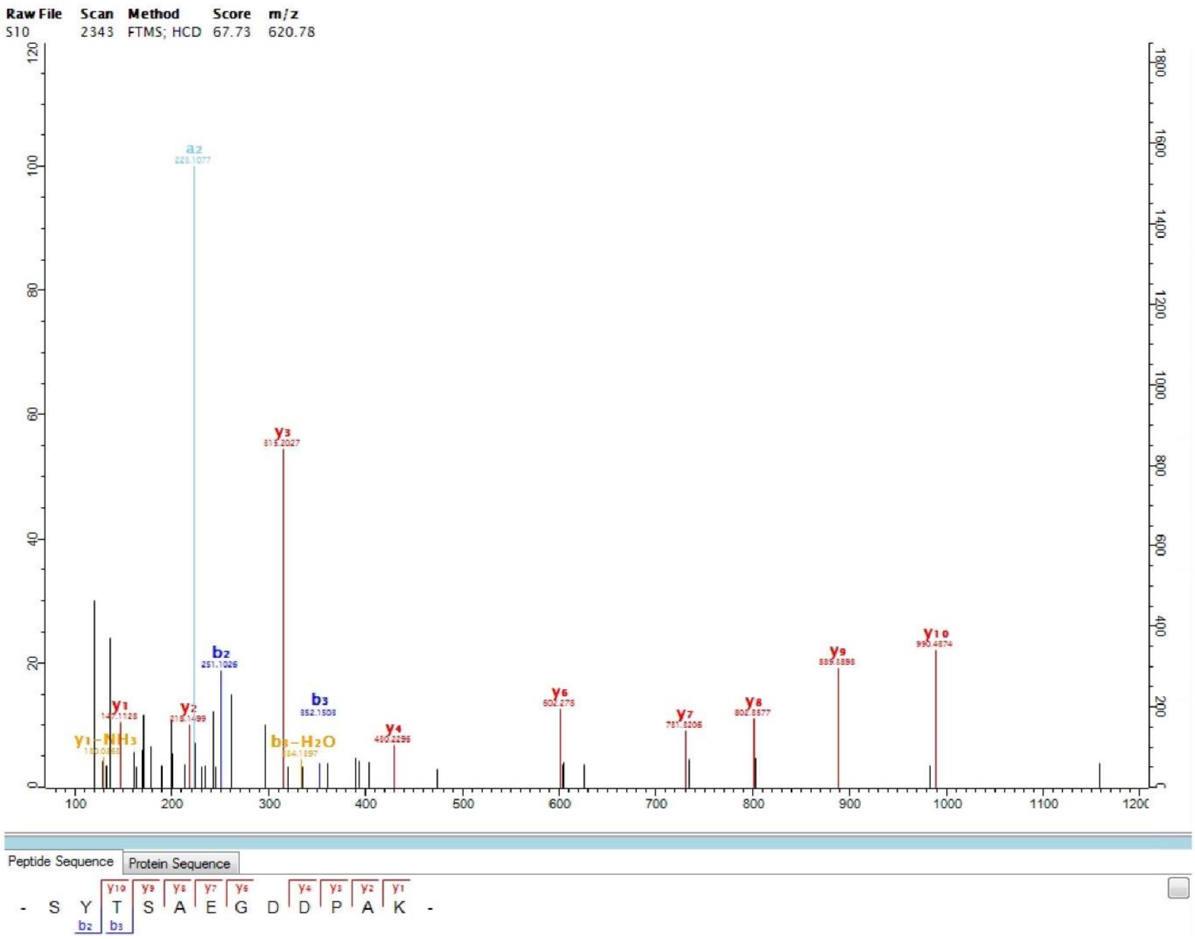

Figure S2. MS/MS spectra of the identified peptide SYTSAEGDDPAK by nano LC-MS/MS.
